# Physicochemical and Antimicrobial Properties of Cocoa Pod Husk Pectin Intended as a Versatile Pharmaceutical Excipient and Nutraceutical

**DOI:** 10.1155/2016/7608693

**Published:** 2016-03-14

**Authors:** Ofosua Adi-Dako, Kwabena Ofori-Kwakye, Samuel Frimpong Manso, Mariam EL Boakye-Gyasi, Clement Sasu, Mike Pobee

**Affiliations:** ^1^Department of Pharmaceutics, Faculty of Pharmacy and Pharmaceutical Sciences, College of Health Sciences, Kwame Nkrumah University of Science and Technology (KNUST), Kumasi, Ghana; ^2^School of Pharmacy, University of Ghana, Legon, Ghana

## Abstract

The physicochemical and antimicrobial properties of cocoa pod husk (CPH) pectin intended as a versatile pharmaceutical excipient and nutraceutical were studied. Properties investigated include pH, moisture content, ash values, swelling index, viscosity, degree of esterification (DE), flow properties, SEM, FTIR, NMR, and elemental content. Antimicrobial screening and determination of MICs against test microorganisms were undertaken using agar diffusion and broth dilution methods, respectively. CPH pectin had a DE of 26.8% and exhibited good physicochemical properties. Pectin had good microbiological quality and exhibited pseudoplastic, shear thinning behaviour, and high swelling capacity in aqueous media. The DE, FTIR, and NMR results were similar to those of previous studies and supported highly acetylated low methoxy pectin. CPH pectin was found to be a rich source of minerals and has potential as a nutraceutical. Pectin showed dose-dependent moderate activity against gram positive and gram negative microorganisms but weak activity against* Listeria* spp. and* A. niger*. The MICs of pectin ranged from 0.5 to 4.0 mg/mL, with the highest activity against* E. coli* and* S. aureus* (MIC: 0.5–1.0 mg/mL) and the lowest activity against* A. niger* (MIC: 2.0–4.0 mg/mL). The study has demonstrated that CPH pectin possesses the requisite properties for use as a nutraceutical and functional pharmaceutical excipient.

## 1. Introduction

Cocoa or* Theobroma cacao* L. (family: Sterculiaceae) is an important agricultural and economic crop which grows in several tropical areas such as West Africa, South America, and Central America [1, 2]. Cocoa beans are the primary economic part of the cocoa fruit and are the main ingredients in the manufacture of chocolate. In West Africa, cocoa is extensively cultivated in many countries, with Cote d'Ivoire and Ghana being the first and second largest producers of cocoa beans in the world, respectively. In Ghana, cocoa cultivation offers employment to about 800,000 farm families and generates about $2 billion annually in foreign exchange, and it is a major contributor to the gross domestic product [3].

The recovery of cocoa beans from the cocoa fruit generates large amounts of waste in the form of cocoa pod shells or cocoa pod husks (CPHs) estimated at 52–76% of the cocoa fruit [4–6]. In fact, a ton of dry cocoa beans produced generates approximately ten tons of CPH [7, 8] and the waste generally remains underexploited [1, 5]. After harvesting the beans, CPHs are traditionally left as undesirable waste to rot in the cocoa farms and plantations, constituting an environmental menace and presenting a challenging waste management problem. With the increasing demand for cocoa beans to satisfy the increasing demand for chocolates, it is anticipated that the production of CPH will continue to increase in the years ahead. Decomposing CPH waste, apart from producing foul odours in the cocoa farms and plantations, is a carrier of botanical diseases such as black pod rot [5, 7–9].

An economical and environmentally friendly way of dealing with the CPH waste menace is to process them into pectins which are natural polymers containing linear chains of (1, 4)-linked *α*-d-galacturonic acid residues, with methyl esters of uronic acid [10]. The composition of pectin is influenced by the botanical source, method of extraction, and environmental factors [11]. For instance, pectin extracted from citrus has less neutral sugars and smaller molecular size compared to pectin from apples [12]. Pectins are versatile naturally occurring polysaccharides with wide and innovative applications in the pharmaceutical, cosmetic, and food industries. In the pharmaceutical industry, pectins are employed as excipients in the manufacture of emulsions, suspensions, matrix tablets, film-coated tablets, compression coated tablets, extended release dosage forms, and colonic delivery dosage forms [13–16].

Various techniques and solvent systems such as water, citric acid, hydrochloric acid, and nitric acid have been employed in the recovery and extraction of pectins from CPH with varying levels of success, with respect to yield and quality of pectin extracted [1, 4, 17–20]. The uses of water and citric acid in the extraction process are, however, more appealing because of their safety and environmental friendliness.

Although considerable research has been devoted to the development of pectin from commercial sources, such as apple pomace and citrus peel, with versatile functional properties in pharmaceutical applications, little attention has been paid to the pharmaceutical applications of CPH pectin. The objective of the present study was to evaluate the physicochemical properties, elemental composition, and antimicrobial properties of CPH extracted with water and citric acid. It is envisaged that results from this study would help in determining the suitability or otherwise of CPH pectin as a potential functional pharmaceutical excipient and nutraceutical.

## 2. Materials and Methods

### 2.1. Materials

Sodium hydroxide (UK), gelatin and lead acetate (France), ferric chloride (India), hydrochloric acid and Mayer's reagent (England), and Dragendorff's reagent and Marquis reagent (England) were purchased. Mannitol salt agar, MacConkey agar, Bismuth sulphite agar, Cetrimide agar, Sabouraud dextrose agar, nutrient agar, potato dextrose agar, and nutrient broth were obtained from Oxoid (England). Ciprofloxacin powder (batch number AV 4008, Maxheal Labs Pvt. Ltd., India), Amoksiklav powder (Amoxicillin + clavulanic acid) (Lot EN 2737, Lek Pharmaceuticals, Slovenia), and Nystatin (100,000 IU/drop, Egyptian Pharmaceutical Industries, Egypt) were used. All other chemicals used were of analytical grade.

Two typed cultures,* Staphylococcus aureus* NCTC7972 and* Escherichia coli* NCTC5933, and seven clinical strains,* Bacillus subtilis* KBTH2014,* Pseudomonas aeruginosa* KBTH2014,* Salmonella typhi* NMIMR 2014,* Shigella* spp. NMIMR2014,* Enterococcus* spp. NMIMR2014,* Listeria* spp. NMIMR2014, and* Aspergillus niger* NMIMR2014, were used as test microorganisms.

### 2.2. Collection and Extraction of CPH Pectin

Ripe mature cocoa pods were harvested from* Theobroma cacao* L. in an experimental plantation of the Cocoa Research Institute of Ghana (CRIG), Tafo, Ghana. The pulp and seeds were removed, and the fresh whole pod husks were peeled to avoid the pigmentation of the skin which may cause longer and more extensive extraction [8, 21, 22]. The peeled husks were minced and prepared for extraction. Pectin was extracted from fresh CPHs according to a previously outlined procedure [1, 23] with minor modifications. Fresh CPHs were minced with a mechanical blender. Hot aqueous and hot aqueous citric acid (4% w/v) extraction of the fresh peeled minced husks (1.05 g/mL) were carried out in a water bath at 50°C. The extract was precipitated with ethanol and filtered twice with two-fold linen cloth. The extract was treated with twice its volume of ethanol and washed thrice to remove impurities. Extraction was repeated to exhaustion and the extract was dried under vacuum. The hot water soluble and hot aqueous citric acid soluble extracts were separately freeze-dried in a freeze dryer (Model 7670520, Labconco, USA) at 0–120 mBar and −41°C and the pectin yield was determined. The freeze-dried pectin samples were stored in aluminium foils in a desiccant at −4°C until used.

### 2.3. Identification Test and Phytochemical Screening of Pectin

One milliliter of 2 N NaOH was added to 5 mL of 1 in 100 solutions of the CPH extract and was allowed to stand at room temperature for 15 minutes. The gel from the preceding test was acidified with 3 N HCl, shaken vigorously, and boiled [24]. Phytochemical screening [25] of CPH pectin was undertaken to determine the presence or otherwise of major phytoconstituents such as tannins, alkaloids, and saponins. Five hundred milligrams of powdered hot water soluble CPH pectin was boiled in 25 mL of water for 5 min. The solution was cooled and filtered and the volume adjusted to 25 mL and used for the phytochemical screening. In the test for tannins, 1 mL portions of the pectin solution were, respectively, added: (a) 10 mL of water and 5 drops of 1% lead acetate solution, (b) a few drops of 1% gelatin solution, and (c) a few drops of 5% ferric chloride solution. The formation of a white precipitate in (a) and (b) and a dark green or deep blue precipitate in (c) indicated the presence of tannins. To test for alkaloids, a few drops of Marquis reagent, Mayer's reagent, and Dragendorff's reagent were separately added to 2 mL portions of the pectin solution. The observation of a colour change (Marquis) and the formation of cream coloured precipitate (Mayer's) and reddish brown precipitate (Dragendorff's) indicated the presence of alkaloids. In the test for saponins, 5 mL of the pectin solution in a test tube was shaken vigorously for 5 min and the formation of stable foam lasting at least 15 min indicated the presence of saponins.

### 2.4. Physicochemical Properties of CPH Pectin

The moisture content was determined by weighing 1 g of CPH pectin into each of three petri dishes and dried in an oven at 105°C to constant weight. The moisture content was determined as the ratio of the weight of moisture loss to weight of sample expressed as a percentage. The pH of 1% w/v solution of hot water soluble pectin and citric acid soluble pectin samples was determined with a calibrated pH meter. The total ash content and insoluble ash residue were determined according to the British Pharmacopoeia method [26]. One gram of pectin sample was weighed and ignited in a furnace at 450°C. The ash obtained was weighed and boiled in 25 mL of 2 M HCl for 5 minutes. The insoluble matter was filtered and washed with hot water and ignited. The subsequent weight was then determined. The swelling index of the pectin sample was determined according to a WHO method [27]. One gram of the sample was weighed into a 25 mL measuring cylinder and the volume occupied was noted (*V*_1_). Twenty-five milliliters of distilled water was added to the sample and shaken intermittently for 1 hour. The sample was allowed to stand for 3 hours and the volume occupied was noted (*V*_2_). The swelling capacity was calculated as follows: swelling capacity = (*V*_2_/*V*_1_) × 100. The degree of esterification (DE) was determined using the acid-base titration method of the Food Chemicals Codex [28].

In the determination of the bulk and tapped densities, 3 g of pectin powder was weighed into a 10 mL measuring cylinder and the volume occupied was noted. The sample was tapped till the powder was consolidated and the volume after tapping was noted. The bulk and tapped densities, as well as the Hausner ratio and compressibility index, were calculated as follows:

(1) Tapped  density=weight  of  pectintapped  volume,Bulk  density=weight of pectinbulk  volume,Hausner  ratio=tapped  densitybulk  density,Carr's  compressibility  index=tapped  density−bulk  densitytapped density×100.

The angle of repose was determined by weighing 10 g of pectin powder into a funnel clamped to a stand with its tip 10 cm from a plane paper surface. The powder was allowed to flow freely onto the paper surface. The height of the cone formed after complete flow and the radius of the cone were used to calculate the angle of repose (*θ*). Consider tan⁡*θ* = *H*/*R*, *θ* = tan^−1^⁡(*H*/*R*), where *H* is the height of the heap and *R* is the radius of the heap. The viscosity of 5% w/v aqueous solution of cocoa pectin was determined at room temperature (25°C) after heating to 33°C, using a Brookfield viscometer (LVT). Determinations were made using spindle 61, by varying the shear rate. Readings on the dial of the viscometer were multiplied by the conversion factor and the results were recorded.

### 2.5. Scanning Electron Microscopy (SEM) Studies

Specimens of hot water soluble pectin and citric acid soluble pectin were prepared for SEM analysis with a thin coating of colloidal carbon for electron conductivity. The morphological features of the samples were studied with a scanning electron microscope (Hitachi S3200N, Japan), using EDAX Genesis. All imaging was viewed under conventional high-vacuum mode and secondary electron scintillator detection mode.

### 2.6. FTIR, NMR, and Elemental Analysis

A sample of hot water soluble pectin and citric acid soluble pectin was analysed for main functional groups using Bruker Alpha Fourier transform infrared spectrophotometer (Germany) operating on Platinum-ATR to obtain FTIR spectra at 400–4000 cm^−1^. Specimens of hot water soluble and citric acid soluble pectin were prepared for NMR analysis using a Varian 500 NMR spectrometer (USA). The ^13^C NMR spectra of the hot water soluble pectin and citric acid soluble pectin extracts in D_2_O were obtained at 25°C and 50°C, respectively. Chemical shifts were expressed in *δ* (ppm) relative to acetone for hot water soluble pectin (*δ* 30.16) and citric acid soluble pectin (*δ* 29.65). The results were analysed by MestReNova NMR. In the elemental analysis, pellets of hot water soluble pectin were prepared and irradiated with an energy dispersive X-ray fluorescence spectrometer (Spectro X-Lab 2000, Kleve, Germany). Peaks shown by the spectrometer indicated the presence of particular elements, while the area under the peaks was an indication of the quantity of elements present.

### 2.7. Evaluation of Microbiological Quality of CPH Pectin

Profiling of possible microbial contaminants from cocoa pectin was undertaken [26] and the microbial load (total aerobic viable count) of pectin per the plate viable count was determined. Dilutions of pectin sample (1 : 10) were done serially to a sample dilution of 10^8^. One mL aliquot of each dilution was transferred aseptically into 20 mL of molten nutrient agar and the plates were allowed to set. The plates were inverted and incubated for up to 48 hours and pure colony forming colonial (cfu) counts were estimated. The presence of the following pathogenic microbes,* E. coli*,* S. aureus*,* Salmonella* spp.,* P. aeruginosa*, and yeasts and moulds in pectin was evaluated. A 1 : 10 dilution of pectin in sterile water was introduced into the primary medium which was nutrient broth. 1 mL of grown culture of the test organism was introduced into the appropriate culture medium in the molten state at 42°C and stabilised at 28°C. The seeded culture media were incubated at 37°C, with an incubation time of 24–72 hours.

### 2.8. Antimicrobial Screening of CPH Pectin by Agar Diffusion

Four concentrations (1.25, 2.5, 5.0, and 10.0 mg/mL) of hot water soluble CPH pectin and standard antibacterial agents, Amoksiklav and ciprofloxacin, as well as the antifungal Nystatin, were used to assess their comparative antimicrobial activities by agar diffusion method [26]. Twenty (20) mL aliquots of molten nutrient agar (antibacterial test) and potato dextrose agar (antifungal test) were melted at 42°C and stabilised at 28°C and aseptically seeded with 0.1 mL of 24 h broth cultures of the appropriate test organisms, poured into sterile petri dishes, and allowed to solidify in a laminar flow chamber. A 10 mm diameter cork borer was used to create four ditches in the set agars. Alternate holes were filled with the exact volumes of aqueous solution of the extracts. Positive and negative test controls were set up alongside the test extract. All plates were left in the chamber for an hour to allow for diffusion. The nutrient agar seeded plates were inverted and incubated at 37°C for 18 hours, while the dextrose agar seeded plates were incubated at room temperature (25°C) for 72 hours. Zones of growth inhibitions due to the activity of the extract and the commercial antimicrobial agents were measured after the incubation periods and recorded.

### 2.9. Determination of Minimum Inhibitory Concentration (MIC)

The MIC of hot water soluble CPH pectin was determined using the broth dilution technique. Graded concentrations of pectin (0.125, 0.25, 0.5, 1.0, 2.0, 4.0., and 8.0 mg/mL) in nutrient broth and potato dextrose liquid medium were compared to those of Amoksiklav, ciprofloxacin, and Nystatin. A set of seven double strength nutrient broth tubes were arranged from a prepared stock solution of 50 mg/mL pectin test sample. Volumes of the stock solution required to produce the respective concentrations with the double strength nutrient broth were calculated and added aseptically to the broth by means of sterile syringes in a laminar flow chamber. The volumes of sterile distilled water required to make the broth tubes single strength were also calculated for and added to the broth tubes aseptically. Finally, 0.1 mL inoculum of a 24 h test microbial culture was inoculated into the broth to complete the procedure. Uniform mixing was ensured and the tubes were incubated at 37°C for 24 h. The tubes were observed for growth (turbidity) after the incubation period and MICs for pectin and the standard antimicrobial agents were determined.

## 3. Results and Discussions

### 3.1. Extraction, Identification, and Phytochemical Constituents of CPH Pectin

The extraction yield obtained from CPHs was 23.30 ± 2.00% and 10.50 ± 0.04% (on dry weight basis) for hot water soluble pectin and citric acid soluble pectin, respectively. Previous reports indicate that pectin was extracted from dried residue pod husk flour [1, 29]. However, in the current study, extraction was undertaken using fresh CPHs and repeated to exhaustion to optimize the yield [17] which is sometimes affected by drying and associated enzymatic activity [30, 31]. It has been reported that the major part of hot water soluble polysaccharides of CPHs is pectin [32]. Aqueous extraction of pectin has advantages over extraction with mineral acids as there is no production of corrosive effluents [29]. Citric acid is a natural, safe food additive and more attractive to use than other strong mineral acids used in the extraction of commercial pectins, which could adversely affect the environment [1]. The extractive yield of cocoa pectin is known to vary depending on the extraction conditions employed and recent studies have shown yields as low as 2% and as high as 20% [4, 18, 19, 22, 29]. Identification test carried out on the extracted samples yielded colourless gelatinous precipitates indicating the possible presence of pectin in the two extracts.

Phytochemical screening of CPH yielded polyphenols such as tannins, alkaloids, and saponins. Phenolic compounds of cacao include catechins, epicatechins, anthocyanins, proanthocyanidins, phenolic acids, condensed tannins, other flavonoids, and some minor compounds [33–35]. Polyphenolic compounds usually accumulate in the outer parts of plants, such as shells, skins, and husks [36]. Previous reports have shown that CPH flour is a source of functional compounds such as phenolics, pectins, and fibre which possess good health benefits [29]. Polyphenols offer protection against coronary heart disease, cancer, and neurodegenerative disorders due to their antioxidant and free radical scavenging properties [37].

### 3.2. Physicochemical Properties of CPH Pectin

Table 1 presents some physicochemical properties of cocoa pectin. The pH of a 1% w/v hot water soluble pectin and citric acid soluble pectin was 6.7 and 3.4, respectively. Thus, pH of the aqueous soluble pectin was near neutral, while the high acidity of the citric acid soluble pectin is likely due to the use of citric acid in the extraction process. The moisture content of pectin was very low (0.2%). This is likely to protect the powdered samples from microbial attack and also to improve the mechanical properties of the powders. The level of purity of pectin sample can be determined by its ash value. This value is indicative of the level of adulteration or handling of the sample. The acid insoluble ash value is an index of mineral or extraneous matter present in a sample. High ash values for cocoa pectin in contrast to pectin from other sources have been reported and generally range between 6.7 and 9.8% [18, 22, 29]. However, these varied values could have been affected by the mode of extraction of the sample [18]. In the present study, the acid insoluble ash value of cocoa pectin was 1.0%, in accordance with official specification [24].

The swelling characteristics of cocoa pectin in various media were investigated. The swelling index of cocoa pectin was 274.7 in 0.1 N HCl, 357.3 in phosphate buffer pH 6.8, and 360 in water. Cocoa pectin can swell to varying extents depending on the pH, ionic strength, and presence of salts in the medium. The swelling behaviour of CPH pectin shows that it can function as a binder or matrix agent in controlled release formulations. This is because swelling is an important mechanism in diffusion controlled release in drug delivery [38]. The degree of esterification (DE) of CPH pectin was 26.8%, indicating that it is a low methoxy pectin. This observation is in agreement with earlier reports [1, 29]. The DE determines the behaviour of pectin and influences its mechanism of gelation. Low methoxy pectins have a DE of 20–40%, while high methoxy pectins have a DE of 60–75%. Low methoxy pectins require a controlled amount of calcium or divalent cations to achieve gelation, while high methoxy pectins undergo gelation in the presence of sugar [39].

The precompression parameters of cocoa pectin powder studied were the angle of repose, bulk density, tapped density, Hausner ratio, and Carr's compressibility. The ease of flow of powders is of paramount importance in tablets and capsules formulation as free flowing powders ensure reproducible filling of tablet dies and capsule dosators, thereby improving weight uniformity and consistency in physical properties. Hausner ratio is related to interparticle friction in a powder and values close to 1.2 are indicative of less cohesive and free flowing powder while values greater than 1.6 are powders which are cohesive and have poor flow properties. In terms of flowability, powders with compressibility index of 5–15% are regarded as excellent, 12–16% good, 18–21 fair, and >40% extremely poor. A high angle of repose is indicative of a cohesive powder, while a low angle of repose connotes a noncohesive powder. In general, powders with angles of repose >50° have unsatisfactory flow properties, whereas minimum angles close to 25° have very good flow properties [40]. In the current study, cocoa pectin powder had Hausner ratio of 1.17, compressibility index of 14.58%, and angle of repose of ~38°. These values are indicative of a powder which is less cohesive and has good flow properties.

**Figure 1:**
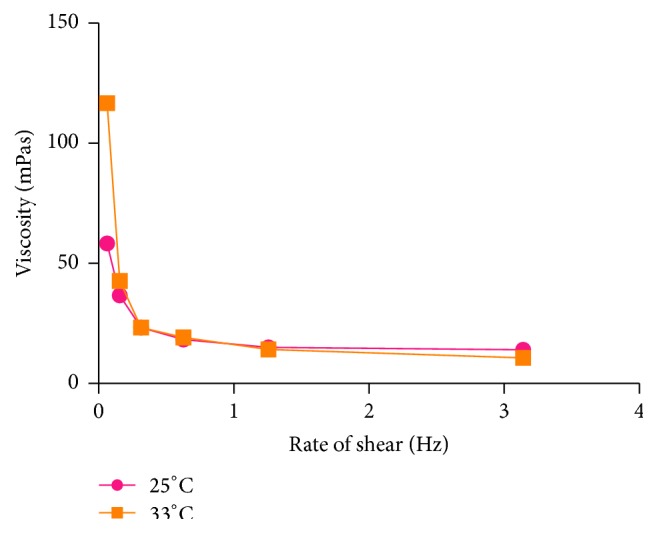
Viscosity profiles of 5% w/v hot water soluble pectin.

The rheograms of 5% w/v hot water soluble cocoa pectin at 25°C and 33°C showed a non-Newtonian, pseudoplastic, shear thinning behaviour (Figure 1). This is similar to earlier reports of other pectin solutions and polysaccharide pharmaceutical excipients [1, 29]. Increasing the temperature of the sample from 25°C to 33°C did not have any marked effect on viscosity of the pectin sample (*p* > 0.05, Student's *t*-test *p* value of 0.59). Although further investigation is necessary, the pseudoplastic behaviour of cocoa pectin is advantageous in its use as a pharmaceutical excipient.

**Figure 2:**
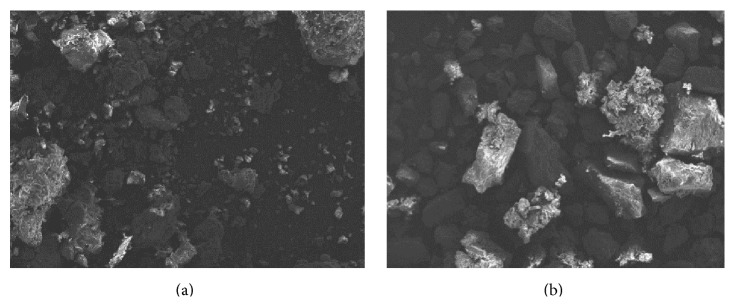
Scanning electron micrographs of (a) hot water soluble pectin (mag ×20) and (b) citric acid soluble pectin (mag ×20).

The scanning electron micrographs of hot water soluble pectin and hot citric acid soluble pectin are shown in Figure 2. The surface characteristics of the samples depict irregular shapes, nonuniform sizes, and rough surfaces. Drug release from a dosage form is affected by the surface characteristics of the excipients used. A rough surface will entrap drug particles in the pores and crevices, resulting in retarded drug release. Hot water soluble and hot citric acid soluble pectin would be able to sustain drug release due to the rough surface exhibited [41]. Both powders contained large to fine particle sizes. Fine particles have a tendency of filling the voids between the larger ones and help to reduce the bulkiness of the powder. Also, the dissolution rate of polysaccharide powders tends to increase with the reduction in particle size [42, 43].

**Figure 3:**
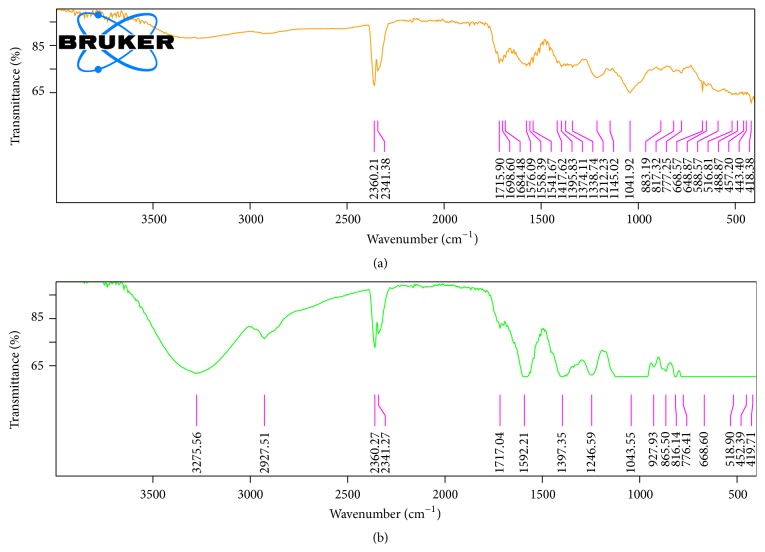
FTIR spectra of (a) citric acid soluble pectin and (b) hot water soluble pectin.

Figure 3 shows the FTIR spectra of CPH pectin extracted with different solvents. The two spectra are identical and showed similar functional groups. Data obtained is indicative of –OH stretching absorption bond. Alcohols show a conspicuous –OH stretching absorption bond at 3000–3700 cm^−1^, which could be narrow or broad depending on whether it is free or involved in hydrogen bonding. The band observed between 2800 and 3100 cm^−1^ is typical of sp3-C–H stretch. Moreover, there was a prominent band between 1000 cm^−1^ and 1200 cm^−1^ which was indicative of a typical C–O stretch such as a glycosidic linkage. The band appearing at 1716 cm^−1^ was typical of a carbonyl group and another band at 1596 cm^−1^ suggests a carboxylate, a salt of a free acid. Furthermore, the band at 2360 cm^−1^ suggests an S–H or C–S bond.

**Figure 4:**
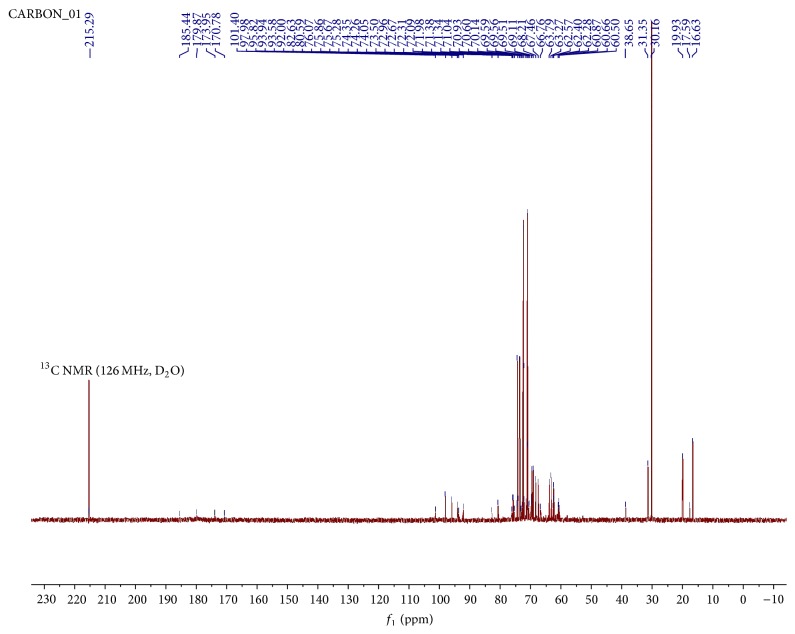
^13^C NMR spectrum of hot aqueous extract of pectin at 25°C in D_2_O.

The ^3^C NMR spectrum of hot water pectin is shown in Figure 4. Chemical shifts were expressed in *δ* (ppm) relative to acetone (*δ* 30.16). Chemical shifts due to anomeric carbons were identified in the range *δ* 95.82–101.4. Signals at *δ* 101.4 and *δ* 97.98 were assigned to C-1 of esterified and nonesterified units of *α*-galacturonic acids, respectively. Previous work showed these signal shifts at *δ* 100 and 99.3 [1] and *δ* 100.1 and 99.4 [19], respectively. This is also similar to earlier reports of signals of C-1 of low methoxy pectins in the range *δ* 101.03–101.43 [44] and of anomeric carbon of *α*-linkage [45]. Signals observed in the region, *δ* 170.78, 173.95, 179.87, and 185.44, were assigned to carbonyl groups of the esterified and nonesterified units [46, 47]. C-6 methylated carbonyl signal shifts were identified at *δ* 170.78 and carboxylic acid signals were identified at *δ* 173.95. Previous report showed similar high frequency C-6 signals at *δ* 170.6 and 173.4, respectively [19]. Signals at *δ* 17.59 were attributed to methyl carbons of rhamnose residues [44]. Signals attributed to C-2, 3, 4, and 5 in the galacturonic acid units were found in the range *δ* 66.76–80.59. C-4 signal shifts of galacturonic acid units were identified at *δ* 70.14–71.38 and *δ* 80.59. Previous work showed signals in the range *δ* 70-71, with substituted residues with C-4 shifts at *δ* 77–79, indicating (1, 4) glycosidic linkages in the homogalacturonan region [47]. In the anomeric region, C-1 rhamnose shift was detected at *δ* 95.82, with the methyl carbon at *δ* 16.63. Chemical shifts at *δ* 19.93 were assigned to methyl groups from the acetyl group [1, 47]. Signals at *δ* 215.29 were attributed to a carbonyl group from the aldehyde or ketone of the reducing sugar. The data above supports the structure of a highly acetylated low methoxy pectin.

**Figure 5:**
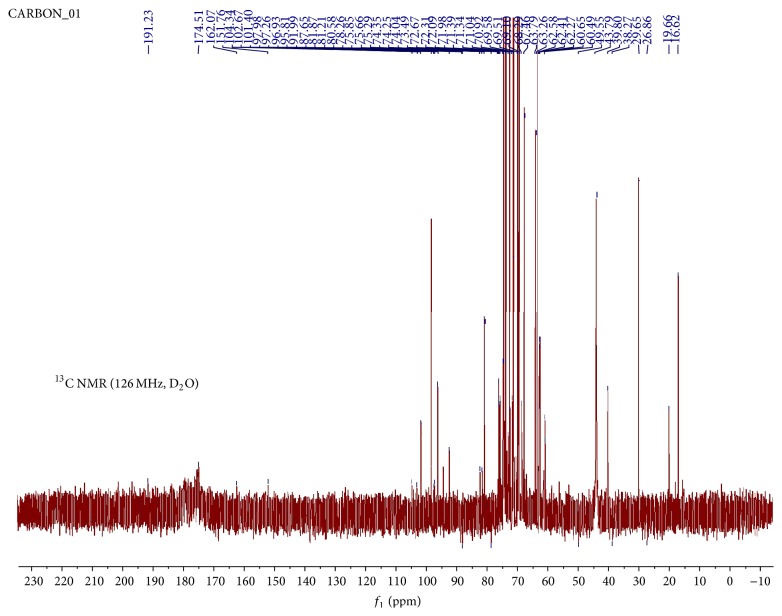
^13^C NMR spectrum of citric acid soluble pectin at 50°C in D_2_O.

Figure 5 shows the ^13^C NMR spectrum of citric acid soluble pectin. Chemical shifts of citric acid soluble pectin were expressed in *δ* (ppm) relative to acetone (*δ* 29.65). Signal shifts attributed to carbons from citric acid were seen at *δ* 39.80. Signals at *δ* 101.4 and *δ* 97.98 were assigned to C-1 of esterified and nonesterified units of galacturonic acids. Previous work showed these signal shifts at *δ* 100 and 99.3, respectively, from methyl ester carbonyl carbons of esterified and nonesterified units from a homogalacturonan [1]. Signal shifts of *δ* 97.26 were attributed to C-1 rhamnose units with the methyl group at *δ* 16.62 [46]. Previous reports identified signals at *δ* 98.5 and 16.6, respectively [1, 19]. Methyl carbons of acetyl groups were seen at a signal shift of *δ* 19.66. Earlier reports showed the signal at *δ* 20.5 with C-6 carbonyl carbon of the acetyl group at *δ* 174.51 [47]. The C-1 signal shifts of *δ* 102.67 were assigned to C-1 of the anomeric region of substituted and nonsubstituted galacturonic acid units, further supporting *β*-linkage [45]. Previous reports showed signals at *δ* 103.3 and *δ* 102.4, of *β* 1, 4-D galacturonic acid units. Aromatic carbon signal shifts were observed at *δ* 151.76 indicating the presence of phenolics [1]. The NMR data shows the presence of highly acetylated pectins with low methoxy groups [1]. The two pectin extracts basically had the same chemical structure which is in accordance with published reports [1, 19].

Results of the elemental analysis of hot water soluble CPH pectin are shown in Table 2. Some major elements or macrominerals identified were sodium, magnesium, calcium, iron, potassium, phosphorus, and sulphur. For the major elements, K was the predominant element (2.269%) followed by Mg (0.219%), P (0.096%), and S (0.094%). Minor elements or microminerals found in cocoa pectin include chromium, copper, zinc, and cobalt. The highest concentration of the minor elements was Cu (10.90%) followed by Zn (8.30%) and Ni (3.70%). Minerals are inorganic substances usually in trace amounts required for the normal functioning of the body. They are involved in bones and teeth development and regulation of metabolic processes of the body by acting as cofactors for enzymes and as catalysts for cell reactions.

Previous reports show that CPH flour also contained a variety of minerals. The qualitative components are similar to those reported in previous studies [5]. In one study, a predominance of K was observed, followed by Ca and Mg, with intermediate proportions of Na, Fe, Mn, and Zn and minor amounts of Cu and Se [29]. Another study reported Ca and K as the major elements [18], while an African cocoa pod husk was found to contain K (3.18%), Ca (0.32%), and P (0.15%) as the major elements [48]. The minerals content of CPH pectin affects both the viscosity and the swelling capacity of the polymer in aqueous media.

The wide range of macro- and microminerals found in cocoa pectin shows the potential of this natural polymer to provide medical or health benefits users. Cocoa pectin is therefore a potential plant-based nutraceutical. There is a growing interest in the use of plant-derived bioactive compounds in foods as “multifunctional food additives” due to their additional nutritional and therapeutic effects [49, 50]. Other plant-based bioactive materials with demonstrable nutraceutical properties include citrus fruits, modified citrus pectin, and apple pectin [51–54]. Plant polysaccharides such as cocoa pectin are generally nontoxic, chemically stable, readily available and renewable, and a rich source of macro- and micronutrients. These polysaccharides are under extensive investigation as potential excipients for the formulation of solid and liquid dosage forms and also as a nutraceutical.

### 3.3. Microbial Quality and Antimicrobial Properties of CPH Pectin

In general, microbial contaminants may be grouped into harmful, objectionable, and opportunistic organisms. Harmful organisms are toxins-producing, disease causing organisms such as* S. typhi*,* E. coli*,* P. aeruginosa*, and* S. aureus*. Objectionable organisms can cause disease or may interrupt the function of the agent leading to the deterioration of the product. These include* Salmonella* species (proteolytic types) and fungi (mycotoxin producing types),* Pseudomonas* spp., and* Candida albicans*. Organisms are said to be opportunistic if they produce disease or infection under special environmental conditions. Harmful organisms are excluded from all pharmaceutical products and excipients. With regard to the CPH pectin sample tested, no harmful microorganisms were identified (Table 3) and the total microbial count was within the specified limit; hence, it passed the microbial quality test [26].

The antimicrobial activity of CPH pectin against selected microbial strains is presented in Table 4. The activity of CPH pectin against test microbial strains indicated by the zones of growth inhibitions was interpreted as follows: ≥30 mm, exceptionally active; 25–30 mm, active; 20–25 mm, moderately active; 15–20 mm, slightly active; <15 mm, peripheral/weak activity [55]. CPH pectin showed dose-dependent moderate antibacterial activity in concentrations of 1.25–10 mg/mL against* S. aureus, P. aeruginosa, B. subtilis, E. coli, Salmonella typhi, and Shigella* spp. and slight activity against* Enterococcus* spp. and* Aspergillus niger*. It however showed weak activity against* Listeria* spp. in concentrations up to 10 mg/mL. On the other hand, Amoksiklav and ciprofloxacin showed active and exceptionally active activity, respectively. Generally, CPH pectin exhibited better activity against gram negative than gram positive bacteria.

Table 5 presents a comparative analysis of the MICs of CPH pectin and three standard antimicrobial agents on selected bacteria and fungus strains. For the three gram positive bacteria tested, cocoa pectin had the lowest MIC hence the highest activity against* E. coli*, while the MICs of ciprofloxacin were generally lower than that of cocoa pectin against the test organisms. The MIC of cocoa pectin was lower for* S. aureus* than* B. subtilis*; hence, pectin is more active against* S. aureus*. Also, the MICs of Amoksiklav were lower than that of pectin against the gram positive bacteria tested. The MIC of cocoa pectin against* A. niger* was four times higher than that of Nystatin, the standard antifungal product. The study has demonstrated that cocoa pectin has some activity against all the tested microorganisms. However, the antimicrobial activity was generally lower than that of the three standard antimicrobial agents compared.

The antimicrobial activity of CPH extract was assessed recently against* S. aureus*,* S. epidermidis*,* B. subtilis* (gram positive),* P. aeruginosa*,* K. pneumoniae*, and* S. cholerae* [55]. The researchers found the extract to be ineffective up to 10 mg/mL against the gram positive bacteria tested, but it showed activity against* P. aeruginosa*,* S. choleraesuis*, and* S. epidermidis*. The strongest antibacterial activity was shown against* S. choleraesuis* (MIC: 1.0 mg/mL) and* S. epidermidis* (MIC: 2.5 mg/mL) and the bioactive fractions of the extract were found to be phenols, steroids, or terpenes [56]. The antimicrobial properties of cocoa phenolics against some food bacterial pathogens and certain cariogenic bacteria have also been reported [57]. In that study, the activity of cocoa phenolics was directly correlated with the ability of the chemical substances to penetrate the bacterial cell wall [33, 57]. In view of the antibacterial properties observed, cocoa pectin has the potential to be developed as an antimicrobial agent for extended release natural products and in food preservation [58].

## 4. Conclusion

It can be concluded from the study that cocoa pectin has the requisite microbial quality and physicochemical parameters to be employed as a multifunctional excipient in the pharmaceutical, food, and allied industries. The elemental content analysis showed the presence of a broad range of micro- and macronutrients in cocoa pectin, making it a potentially useful health promotion polymer. Cocoa pectin showed moderate activity against selected gram positive and gram negative bacteria and could be useful as a preservation agent in pharmaceutical formulations and food products. The study has demonstrated the enormous potential of cocoa pectin as a pharmaceutical excipient, a nutraceutical agent, and an antibacterial agent.

## Figures and Tables

**Table 1:** Some physicochemical properties of hot water soluble CPH pectin.

Parameter	Value
Yield on extraction (%)	23.3 ± 2.00
	10.5 ± 0.04^*∗*^
Moisture content (%)	0.19 ± 0.06
Ash value (%)	1.0
pH (1% w/v @ 25°C)	6.73 ± 0.06
	3.43 ± 0.06^*∗*^
Swelling index	
0.1 M HCl	357.3 ± 4.6
Phosphate buffer pH 6.8	274.7 ± 4.6
Distilled water	360.0 ± 0.0
Degree of esterification (%)	26.8 ± 2.5
Precompression properties	
Bulk density (g/mL)	1.881
Tapped density (g/mL)	2.200
Hausner ratio	1.17
Compressibility index (%)	14.58
Angle of repose (°)	37.97

^*∗*^Citric acid soluble pectin.

**Table 2:** Elemental content of hot water soluble CPH pectin.

Type of element	Content (%)
Macroelements	
Na	>0.038
Mg	0.219
P	0.096
S	0.094
K	2.269
Ca	0.011
Fe	0.024
Microelements	
Cr	<0.0006
Co	<1.90
Ni	3.70
Cu	10.90
Zn	8.30
Ga	0.70
Mo	<0.9

**Table 3:** Microbial quality of hot water soluble CPH pectin.

Test protocol	Results	Inference
Total aerobic viable count of sample (BP 2007 specification: ≤1 × 10^5^ cfu/mL)	1.2 × 10^1^ cfu/mL	Passed
Test for *Escherichia coli*, MCA/37°C/48 h (BP 2007 specification: nil)	None detected	Passed
Test for *Staphylococcus aureus*, MSA/37°C/48 h (BP 2007 specification: nil)	None detected	Passed
Test for *Salmonella* spp., BSA/37°C/48 h (BP 2007 specification: nil)	None detected	Passed
Test for *Pseudomonas aeruginosa*, CA/37°C/48 h (BP 2007 specification: nil)	None detected	Passed
Test for *yeasts and moulds*, SDA/25°C/5 days (BP 2007 specification: ≤1.0 × 10^4^ cfu/mL)	None detected	Passed

MCA = MacConkey agar; MSA = Mannitol salt agar; BSA = Bismuth sulphite agar; CA = Cetrimide agar; SDA = Sabouraud dextrose agar.

**Table 4:** Antimicrobial properties of hot water soluble CPH pectin and standard antimicrobial agents against test organisms.

Organisms	Mean zones of inhibition (mm)			
	10 mg/mL	5 mg/mL	2.5 mg/mL	1.25 mg/mL
Gram negative bacteria				
*Escherichia coli*	26.0 ± 0.5	25.0 ± 1.0	22.5 ± 0.5	20.0 ± 0.0
	35.0 ± 0.0^a*∗*^	32.0 ± 1.0^a*∗*^	30.9 ± 0.1^a*∗*^	29.0 ± 0.0^a*∗*^
	37.0 ± 1.0^a*∗∗*^	34.9 ± 0.9^a*∗∗*^	30.0 ± 1.0^a*∗∗*^	26.9 ± 0.9^a*∗∗*^
*Pseudomonas aeruginosa*	24.0 ± 0.5	23.2 ± 0.2	22.0 ± 0.0	19.4 ± 0.6
	36.9 ± 0.1^a*∗*^	35.0 ± 1.0^a*∗*^	30.0 ± 1.0^a*∗*^	27.8 ± 0.8^a*∗*^
	38.9 ± 0.8^a*∗∗*^	37.8 ± 0.8^a*∗∗*^	32.0 ± 0.0^a*∗∗*^	30.0 ± 0.0^a*∗∗*^
*Salmonella typhi*	25.0 ± 1.0	23.2 ± 0.2	20.5 ± 0.5	18.0 ± 0.0
*Shigella* spp.	22.0 ± 0.0	20.3 ± 0.8	18.0 ± 0.0	16.0 ± 0.0
Gram positive bacteria				
*Staphylococcus aureus*	24.0 ± 0.0	22.5 ± 0.5	19.6 ± 0.6	17.0 ± 0.0
	33.9 ± 0.1^b*∗*^	27.3 ± 0.6^b*∗*^	25.0 ± 0.0^b*∗*^	22.0 ± 1.0^b*∗*^
	36.0 ± 0.0^a*∗∗*^	33.9 ± 0.9^a*∗∗*^	29.9 ± 0.1^a*∗∗*^	24.7 ± 0.6^a*∗∗*^
*Bacillus subtilis*	24.0 ± 0.0	22.5 ± 0.5	20.0 ± 0.5	17.0 ± 0.0
	36.0 ± 0.0^a*∗*^	30.9 ± 0.2^a*∗*^	28.0 ± 1.0^a*∗*^	25.0 ± 1.0^a*∗*^
	38.0 ± 0.0^a*∗∗*^	33.0 ± 1.0^a*∗∗*^	24.9 ± 0.9^b*∗∗*^	20.1 ± 0.9^b*∗∗*^
*Enterococcus* spp.	18.0 ± 0.5	16.0 ± 0.0	15.0 ± 0.0	12.7 ± 0.3
*Listeria* spp.	15.0 ± 0.0	13.0 ± 0.0	12.0 ± 0.0	ND
Fungus				
*Aspergillus niger*	18.0 ± 0.7	16.3 ± 0.4	15.0 ± 0.0	ND
	20.3 ± 0.4^b*∗∗∗*^	18.5 ± 0.0^b*∗∗∗*^	17.0 ± 0.0^b*∗∗∗*^	15.1 ± 0.1^*∗∗∗*^

^*∗*^Amoksiklav; ^*∗∗*^ciprofloxacin; ^*∗∗∗*^Nystatin; ND = not determined; ^a^statistically different from pectin (*p* < 0.05); ^b^not statistically different from pectin (*p* > 0.05).

**Table 5:** Minimum inhibitory concentrations (MICs) of CPH pectin and standard antimicrobial agents against test organisms.

Organisms	MIC (mg/mL)			
	CPH pectin	Amoksiklav	Ciprofloxacin	Nystatin
Gram negative bacteria				
*Escherichia coli*	0.5–1.0	ND	0.125–0.250	ND
*Pseudomonas aeruginosa*	1.0–2.0	ND	0.500–1.000	ND
*Salmonella typhi*	1.0–2.0	ND	0.250–0.500	ND
Gram positive bacteria				
*Staphylococcus aureus*	0.5–1.0	0.25–0.50	ND	ND
*Bacillus subtilis*	1.0–2.0	0.50–1.00	ND	ND
Fungus				
*Aspergillus niger*	2.0–4.0	ND	ND	0.5–1.0

ND = not determined.
